# Supplementary material for: Parameter optimization for MINFLUX microscopy enabled single particle tracking
Source: Commun Biol. 2025 Nov 14;8:1573. doi: 10.1038/s42003-025-09060-1 (PMC12618476; doi:10.1038/s42003-025-09060-1)
Supplement: Supplementary file 1 — Supplemental Material [file 42003_2025_9060_MOESM1_ESM.pdf]

Supplementary Table 1 – Complete Experimental Results Reveal Optimization Tradeoff Between Trackable Diffusion Rates and Localization Error for MFX-enabled Single Particle Tracking – In addition to the ideal CRLB $\sigma_{CRLB}(\bar{r} = \bar{0})$ (see Supplementary Material of [8], p.11) we show the apparent lateral diffusion coefficients D_{eMSD} (ensemble average) and D_{tMSD} (time average), as well as the lateral localization error σ_{eMSD} extracted from the ensemble-average Mean-Squared-Displacement (*e*MSD) and time-average Mean-Squared-Displacement (*t*MSD) curve respectively using Optimal Least Squares Fitting (OLSF) while assuming momentary Brownian motion. Here η refers to the number of *cycles*, t_{loc} is the time-to-localization, N is the number of photons, N_{PL} is the *PL* and ⟨… ⟩ the scale-appropriate average value. We indicate the sample size n, i.e. the number of tracks per set, in the last column. The first column S indicates the sample in reference: Dye on GUV-patch SLB (I), QDs on GUV-patch SLB (II), QDs on Lipid-Deposition-SLB (III).

S	PL	DMP	t_{dwell}	D_{eMSD} [$\mu m^2/s$]	D_{tMSD} [$\mu m^2/s$]	$\frac{D_{eMSD}}{D_{tMSD}}$	σ_{eMSD} [nm]	σ_{CRLB} [nm]	$\langle\eta\rangle$	$\langle t_{loc}\rangle$ [µs]	$\frac{\langle N\rangle}{\langle\eta\rangle N_{PL}}$	n
I	10	0	100	2.46	2.51	0.98	30.30	20.03	1	150	2.13	855
II	10	5	100	0.07	0.08	0.85	16.52	20.03	1	150	4.10	924
II	10	2	100	0.49	0.51	0.96	15.53	20.03	1	150	4.49	988
II	10	0	100	0.63	0.77	0.81	28.02	20.03	1	150	3.36	1075
II	10	0	100	0.63	0.78	0.81	27.96	20.03	1	150	3.36	1061
II	20	0	100	0.52	0.61	0.84	22.78	14.35	1	151	2.27	841
II	50	0	100	0.48	0.60	0.80	14.05	9.08	2	282	0.71	534
III	10	0	100	0.46	0.61	0.77	30.37	20.03	1	150	1.53	576
III	20	0	100	0.36	0.46	0.78	23.51	14.35	2	281	0.67	325
III	50	0	100	0.23	0.47	0.50	13.46	9.08	5	676	0.22	323
II	50	0	50	0.28	0.40	0.70	4.67	9.08	5	437	0.23	779
II	50	0	100	0.28	0.33	0.85	5.50	9.08	3	414	0.42	763
II	50	0	200	0.21	0.22	0.93	10.91	9.08	2	483	0.82	564
II	TIRF SPT			0.64	0.63	1.01	39.86	–	1	14925	–	1810

Supplementary Note 1

Overview of the Most Influential Sequence Parameters in MINFLUX enabled Single Particle Tracking

Disclaimer:

The following list of parameters, parameter names, and descriptions given below is subject to change. Any information is provided as is for the MINFLUX-*iMSPECTOR* version *v16.3.15635* supplied by the manufacturer in correspondence to their commercial implementation of MINFLUX microscopy (Abberior Instruments GmbH).

Global Parameters

Automatic Background Estimation

Parameter Name: *bgcSense*

Explanation: Determines if and how many times the background level is gauged during grid search. A nonzero (or true) value indicates that background measurements are performed. When an integer $n > 1$ is provided as input, the background is estimated in increments of n, i.e. at each n-th time that no particle is found within a grid cell, while a value of zero (or false) turns background sensing off.

The background level estimate will be subtracted from the signal obtained in each iteration before the background threshold is applied.

Tip: This is advantageous for heterogeneous samples, e.g. cellular membranes. The Background Threshold should be fit to the sample whether the automatic estimation is used or not. Make sure to use the same value, i.e. the same setting, for *bgcSense* when adjusting the Background Threshold for the sample used.

Expected Input: Integer (typical values within 0–10) or Boolean.

Center Dwell Factor

Parameter Name: *ctrDwellFactor*

Explanation: Controls the fraction of the total dwell time (see *patDwellTime* below) allocated for a CFR-Check. When active, the CFR-Check duration is computed as:

$$t_{center} = ctrDwellFactor \cdot patDwellTime$$

Tip: Keep in mind that the set value determines the time spent in the center spot regardless of the iteration. A value of 1/n, where n equals number of TCP vertices (e.g. 6 for hexagonal), is sufficient to guarantee that only a single fluorophore is detected at a time while minimizing the photon budget consumed by this operation.

Expected Input: Positive Floating-Point Value.

Damping

Parameter Name: *damping*

Explanation: Artificially shortens the update distance between consecutive localizations by a factor:

$$\Delta_{update} = \Delta_{real} \cdot 2^{-damping}$$

Tip: Damping may be used to counter localization overshoot or to mitigate possible jitters introduced by the galvanometric scanner. However, it is discouraged to use during Single Particle Tracking (SPT) as it arbitrarily modifies the results underestimating particle motility (Supplementary Figure 1).

Expected Input: Positive Floating-Point Value.

Localization Limit per continuous Trace

Parameter Name: *locLimit*

Explanation: Sets an upper threshold for the number of valid consecutive localizations allowed along a single trace. The SPT process is stopped after reaching the threshold and returns to the grid search.

Expected Input: Positive Integer or –1 (to disable the limit).

Number of Permitted Localization Attempts

Parameter Name: *stickiness*

Explanation: Defines the number of attempts made to localize a particle. An attempt is terminated by a failed CFR-Check or a break condition triggered by exceeding the *maxOffTime*.

Tip: During SPT it is advantageous to use a permissive sequence that doesn't terminate immediately to improve tracking consistency. Keep in mind that this, however, requires post processing to get rid of mid-trace-particle-swap events.

Expected Input: Positive Integer (with 0 disabling the check).

Sequence Name

Parameter Name: *id*

Explanation: Set a name or identifier for the sequence.

Tip: This is the name that will appear in the *iMSPECTOR* software during sequence selection. It is encouraged to use the filename of the sequence *.json* file for easy readability.

Expected Input: String.

Sequence Repetition Entry Point

Parameter Name: *headstart*

Explanation: Specifies from where to re-enter the sequence after the final iteration has concluded with a particle localization estimated. Positive numbers start counting from the top, negative from the bottom.

Tip: As we aim for speed and photon efficiency during SPT it is highly encouraged to repeat only the final iteration ($headstart = -1$).

Expected Input: Integer.

Local Parameters

Background Threshold

Parameter Name: *bgcThreshold*

Explanation: Dictates the baseline threshold (in Hz) for the background level as a high-pass filter. After each roundtrip, the system determines if the obtained signal can be considered valid foreground. Signal frequencies beyond this value will be considered valid to use for the localization estimation. In case the signal resides below the threshold, another roundtrip is engaged.

Tip: Make sure to adjust the threshold to on a per-experiment basis. Think about the expected sample homogeneity and consider whether to additionally use *Automatic Background Estimation*. Make sure however to stick with the decision and adjust the thresholds of the sequence according to the settings used during tracking.

Expected Input: Positive Integer or –1 (to disable).

Center-Frequency-Ratio Check (CFR Check)

Parameter Name: *CCRLimit*

Explanation: This activates the CFR-Check in the respective iteration when set to a positive value. This means that after finishing the TCP roundtrip, the beam will be placed in the center of the pattern and signal will be integrated for a time dictated by the Center Dwell Factor. After that the Effective Frequency of the Center (EFC) will be compared to the Effective Frequency at the Offset (EFO), i.e. the signal obtained at the vertices, to calculate the CFR:

$$\mathrm{CFR} = \mathrm{EFC} / \mathrm{EFO}$$

If the ratio exceeds the value set for the *CCRLimit*, the track is terminated, and an attempt is concluded.

Tip: It is worth noting that due to the small TCP diameter and the fluorescent target constantly moving, the CFR-Check is not a suitable measure to be used during the tracking of free and or fast particles and should hence be turned off in the final iteration. Additionally, deactivation of the CFR-Check significantly speeds up data acquisition and thus the entire tracking routine. In case the target particle is expected to exhibit sufficiently slow and/or semi-static movement and when sampling speed is not of the essence, it could however be worth considering employing the CFR-Check in the final iteration.

Expected Input: Positive Floating-Point Number or –1 (to disable).

Dwell Time

Parameter Name: *patDwellTime*

Explanation: Specifies the time in seconds the system spends on signal integration on a TCP roundtrip. For each vertex (excluding the extra center spot if the CFR-Check is enabled), the integration time $t_{\mathrm{integrate}}$ is given by:

$$t_{\mathrm{integrate}} = patDwellTime \,/\, N_{\mathrm{vertices}}$$

where $N_{vertices}$ is the number of vertices of the TCP.

Tip: Even in ideal conditions (i.e. $\langle\eta\rangle = 1$) the Dwell Time does not equate the time-to-localization as there is hardware induces temporal overhead $t_{hw}^{\eta=1}$ (compare Equation 7, main text) of about $t_{\mathrm{HEX}}^{\eta=1} \approx 52\mu s$ (Hexagonal Pattern) or $t_{\mathrm{TRI}}^{\eta=1} \approx 35\mu s$ respectively. It is discouraged to choose a Dwell Time that approaches the hardware overhead or goes below it.

Expected Input: Positive Floating-Point Number; corresponding to a time in seconds.

Estimation Coefficients

Parameter Name: *estCoeff*

Explanation: These coefficients provided by the manufacturer (Abberior Instruments GmbH) are used during the localization estimation and correct an initial estimate based on an expected number of photons and a given TCP shape and diameter.

Tip: Make sure to update these coefficients any time the Photon Limit, TCP Diameter and/or TCP Pattern are changed. Some coefficient configurations can be found in the premade iteration blocks called *containers* or be provided by the manufacturer (Abberior Instruments GmbH) upon request. It is possible to vary the Photon Limit slightly for an iteration keeping the same Estimation Coefficients without much decrease in localization performance, though it is encouraged to always use the correct Estimation Coefficients for each iteration to prevent errors and unwanted side effects.

Expected Input: A list of Floating-Point Numbers.

Laser Power Factor

Parameter Name: *pwrFactor*

Explanation: Adjusts the excitation laser power used in the respective iterations by multiplicatively scaling the baseline laser power by the set value.

Tip: It can be used to increase the laser power exclusively during tracking while keeping the photonic stress to a minimum during the rest of the experiment. However, it is advised to keep variation in this value to a minimum and rather adjust the baseline laser power specified in the *iMSPECTOR* software.

Expected Input: Positive Floating-Point Number.

Pattern Repeat

Parameter Name: *patRepeat*

Explanation: Specifies the number of times the TCP is repeated, during a single Dwell Time. The overall sum of the integration time spent at the vertices on all roundtrips is equal to the Dwell Time:

$$patDwellTime = \sum_i t^i_{integrate} = \sum_i patDwellTime \, / \, (N_{\text{vertices}} \cdot patRepeat)$$

Tip: Increasing the repeat count can help counteract issues such as fluorophore blinking or instrument vibrations. Keep in mind however that additional roundtrips add additional hardware overhead to the total time-to-localization (see Dwell Time).

Expected Input: Positive Integer (0 breaks the sequence as no scan is conducted).

Photon Limit

Parameter Name: *phtLimit*

Explanation: Specifies the minimum number of photons required for a valid localization estimation. If not reached within a single TCP roundtrip, another is engaged, provided no other break criterium is triggered (see Stickiness, CFR-Check and Single Interval Linger Time as well as the Methods part of the Main document).

Tip: For larger changes, it is recommended to use the premade iteration blocks taken from the container sequences.

Expected Input: Positive Integer.

Minimum Time Between Localizations

Parameter Name: *Schedule*

Explanation: Specifies a minimum wait time between obtaining a valid position and the next localization attempt. The sample is only illuminated during the localization attempt. This parameter is especially useful for imaging easily bleached fluorophores.

Tip: this parameter can be used for tracking, but only of extremely slow targets, since this additional wait time only makes it easier for faster targets to “escape” the effective TCP area where it can be localized. In general, we recommend using it very sparingly for SPT experiments.

Expected Input: Positive Floating-Point Number; corresponding to a time in seconds.

TCP Diameter L

Parameter Name: *patGeoFactor*

Explanation: Controls the diameter of the TCP by scaling the nominal value ($360nm$). The TCP diameter calculates as follows:

$$L = patGeoFactor \cdot 360nm \cdot (wavelength/642nm)$$

Where *wavelength* is an additional iteration specific reference parameter found in the sequence but excluded in our overview that should not be edited whatsoever.

Tip: It is recommended to use the premade blocks of parameters called *containers* whenever changing the TCP diameter or contacting the manufacturer (Abberior Instruments GmbH).

Expected Input: Positive Floating-Point Number.

TCP Pattern

Parameter Name: *pattern*

Explanation: Defines the TCP scan pattern by selecting one of several preset geometries (such as “hexagon”, “square”, or “triangle”). This directly influences the number of vertices and the overall photon collection geometry.

Tip: It is highly suggested not to change the pattern but to rely on the premade iteration blocks called *containers* provided by the manufacturer (Abberior Instruments GmbH).

Expected Input: String (referencing a preset pattern).

Single Interval Linger Time

Parameter Name: *maxOffTime*

Explanation: Determines a grace interval in seconds that the signal can remain below the Background Threshold during signal integration. This is designed to counteract premature termination of the localization routine due to e.g. flickering of the fluorophore target. When a number of integration roundtrips have passed without collecting the necessary number of photons (see Photon Limit) that in time equal the *maxOffTime* threshold, a break condition is triggered terminating the current attempt.

Tip: Exceeding the *maxOffTime* counts against the Number of Permitted Localization Attempts. It is encouraged to consider the *maxOffTime* as a sort of buffer to counteract unexpected events during tracking and increase consistency and track length as the SPT routine will not be so easily terminated. Keep in mind though that this may require additional post processing to remove e.g. mid-trace-particle-change events or longer lingering times after a particle has been lost and a new one found.

Expected Input: Floating-Point Number. The default “unspecified” equals $3ms$.

Supplementary Note 2

MINFLUX Sequence Optimization Guide for 2D SPT Experiments

The present guide supposes the use of the commercial implementation of MINFLUX (Abberior Instruments GmbH), a reasonable familiarity with the core concepts of MINFLUX microscopy and knowledge on how to appropriately modify the scanning sequences. This knowledge should be acquired before attempting any MINFLUX experiment.

Step 1 Preliminary Questions

Ask yourself the following questions:

- What exactly is the phenomenon of interest?
  - What are the relevant spatial and temporal scales?
  - What is the lateral localization precision necessary to observe it?
- Which is the priority, sampling rate or localization precision?
- Is this an absolute measurement or do I just want to compare different experimental conditions?
- Is the effect I want to observe faster than $50\mu s$?

Note: If the required sampling rate is faster than $20kHz$ ($50\mu s$ localization time), a different approach than MINFLUX is necessary.

Step 2 Fluorescent Label and Optimization of Fluorescence Detection

The goal of this step is to adjust the excitation intensity such that enough photons can be detected in as little time as possible while avoiding photo-damage to the sample as much as possible. A handy guideline is to make sure that the average photon detection rate from the sample, given a known Dwell Time (t_{dwell}) and Photon Limit (PL), is sufficient to obtain the whole photon budget in one iteration. For example, for $PL = 10$ and $dT = 100\mu s$, the target average photon detection rate is at least 100kHz, that is, 10 photons per $100\mu s$.

The size of the emitting particle must be kept in mind as larger targets, e.g. quantum dots, may not be approximated as a point coordinate as the lateral resolution of the microscope is in the same order of magnitude as the spatial extent of the tracked object, which introduces a possible further source of localization error to the measurement.

TIP: Measuring the average photon detection rate of your target at different excitation laser powers may be useful, to evaluate, for example, the influence of photophysical effects such as blinking or flickering of the fluorescent dyes.

Warning: MINFLUX requires extremely low concentrations of target molecules to ensure tracking and reduce the likelihood of mid-trace-target-swap events.

Step 3 Adjust the MINFLUX Sequence

How are MINFLUX Measurements Initiated and Controlled?

Once a suitable ROI is selected in the sample, a MINFLUX measurement can be initiated, and the device will follow a *sequence* containing the instructions to perform it. A MINFLUX sequence is a *.json* file that dictates how the microscope hardware operates once the measurement is started. As the localization routine operates in sequential iterations in *MFX*, there are two types of parameters involved: global ones affecting the entire measurement and local ones affecting only their respective iteration.

General Advice for Modifying MINFLUX Sequence Parameters

When changing the laser power and/or Power Factor parameters, it may be necessary to re-evaluate the background threshold. The same is true when changing the setting for the Automatic Background Estimation (given as *bgcSense* in the sequence file), which can prove useful for highly heterogeneous samples.

When changing *PL* and TCP diameter size (given as *patGeoFactor* in the sequence file), the correct estimation coefficients need to be entered in the sequence files. These are usually provided by the manufacturer (Abberior Instruments GmbH) and can be easily copied into the sequence at the relevant place. If precise estimation coefficients do not exist for the chosen combination of *PL* and *patGeoFactor*, it is possible to choose the next closest combination if the deviation is minimal or reach out to the manufacturer to seek further advice.

Recommendations for Global Parameters

- <u>Disable damping [*damping*: 0] to follow the particle more closely.</u> If severe overshoot is present in the final datasets, or if the measurement is not intended to measure absolute quantities (such as diffusion rates), it is possible to set [*damping*: 1] to avoid possible mechanical overshoots in the scanning hardware when following the particle. It is, however, not recommended to increase it beyond 1, which would result in worse tracking performance.
- <u>Make sure to only repeat the last iteration when tracking by setting [*headstart*: -1].</u> This is essential to ensure fast and reliable tracking.
- <u>Enable permissive tracking [*stickiness*: > 0]</u> to prevent the routine from being disrupted prematurely due to target particle flickering or blinking events. While this may introduce mid-trace-target-swap events it greatly increases the continuity of SPT process. Additionally, these events can be filtered out in post processing with relative ease, given that they introduce a significant temporal gap when they occur.
- (Optional) <u>Turn on the Automatic Background Estimation [*bgcSense*: true]</u> for cell samples. This will enable the MINFLUX to gauge the local background level for each position in the searching phase of the sequence. This is essential for samples with spatially inhomogeneous background.

TIP: Use the *locLimit* parameter to set a maximum number of consecutive localizations per continuous trace. This has two major benefits: first, it will increase the number of different particles tracked by forcing the routine to return to search mode after the localization limit has been reached, and also it reduces the chance of the tracking routine getting "stuck" on of immobile particles that may be present in the sample.

Choosing the TCP size *L* of the Final Iteration

- Fast diffusion, e.g. on SLBs -> L = 150nm [*patGeoFactor*: 0.42]
- Medium-Slow diffusion, e.g. on cells -> L = 100nm [*patGeoFactor*: 0.28]
- Slow diffusion, e.g., molecular motors -> L = 75nm [*patGeoFactor*: 0.21]

Generally speaking, the faster the particle, the larger the L should be. A smaller TCP diameter is especially recommended if optimizing for spatial precision.

Choosing the TCP Geometry for the Final Iteration

- Generally, or in doubt -> Hexagonal pattern
- Only in specific cases, e.g. unusually dim target -> Triangular pattern

It is strongly recommended to always use the hexagonal pattern for tracking as it offers, in principle, superior precision compared to the triangular pattern, in terms of the symmetry of the precision of localization throughout the TCP area. However, restricting the pattern to three vertices increases the average signal integration time per vertex, i.e., TCP scanning position, which may be necessary for fast moving particles with dim fluorescence targets. Due to the reduced number of vertices, a triangular scanning pattern takes about $15\mu s$ less to complete compared to the hexagonal one.

Adjust the Background Threshold

- Adjust your sequence to measure the background by setting the background threshold and photon limit to zero [*bgcThreshold*: 0, *phtLimit*: 0] or pick one of our example sequences.
- Conduct a MINFLUX measurement and estimate a background threshold for the final iteration from the average of the EFO histogram obtained from the *paraFLUX* software provided by Abberior instruments GmbH.

Tip: Do not forget to reset the background threshold and photon limit for all iterations should you want to use the same sequence for tracking later.

Step 4 Engineer the Final Sequence

Initial Conditions

It is recommended to start with a sequence where the final iteration has a permissive photon limit, i.e. with initial parameters that provide fast and reliable sampling with a small time to localization with less emphasis on minimizing the localization error, for example:

- Photon Limit: 10 [*phtLimit*: 10]
- Dwell Time: $100\mu s$ [*patDwellTime*: 100e-6]
- Power Factor: 3 [*pwrFactor*: 3.0]

With such parameters, it is possible to collect the necessary data to further optimize the MINFLUX sequence and tailor it to the experiment.

Tip: Using the Power Factor parameter, it is possible to control the laser power to use during the final iteration. This applies exclusively during the iteration in which it is included, providing the necessary excitation power to produce the desired photon detection rate exclusively during the tracking step, and not before.

Generally recommended settings for MINFLUX SPT experiments

- Disable preset TCP pattern repetitions [*patRepeat*: 1] to reduce the overall average time-to-localization $\langle t_{loc} \rangle$ as each additional repetition adds significant hardware time overhead.
- Disable the Center-Frequency-Ratio Check [*ccrLimit*: -1.0] in the last iteration in the sequence to prevent additional time overhead and significantly reduce the time-to-localization and photonic-stress to the sample by removing an additional detection at the center of the TCP.

Optimization Criteria

Using the thus prepared sequence, take an initial MINFLUX dataset of the labelled sample. Evaluate your data using the tools provided here or your own and further optimize the sequence until ideal conditions are reached. We recommend determining and using the following values to optimize the sequence, whose definition are present in the main text:

Acquisition Speed:

- $\langle \eta \rangle$ – The average number of cycles per localization (Use when adjusting PL)
  To ensure fast and homogeneous tracking, optimize $\langle \eta \rangle = \frac{\langle N \rangle}{\langle \psi_{\mathrm{MFX}} \rangle \cdot t_{dwell}} = 1.$
- $\langle t_{loc} \rangle$ – The average time-to-localization (Use when adjusting t_{dwell})
  To speed up tracking and catch faster particles, optimize $\langle t_{loc} \rangle \rightarrow 0$

Acquisition Fidelity and Photonic Stress:

- $\frac{\langle N \rangle}{\langle \eta \rangle \cdot N_{PL}}$ – Multiples of the photon limit detected per localization
  To ensure fast and homogeneous tracking, optimize $\frac{\langle N \rangle}{\langle \eta \rangle \cdot N_{PL}} > 1$

Where $\langle \psi_{\mathrm{MFX}} \rangle$ is the average photon detection frequency per localization sometimes referred to as EFO, $\langle N \rangle$ $\left(= \langle \psi_{\mathrm{MFX}} \cdot t_{\mathrm{signal}}^{\eta} \rangle = \langle \psi_{\mathrm{MFX}} \cdot \eta \cdot t_{\mathrm{dwell}} \rangle = \langle \psi_{\mathrm{MFX}} \rangle \cdot \langle \eta \rangle \cdot t_{\mathrm{dwell}}\right)$ the average number of collected photons sometimes referred to as ECO, $t_{\mathrm{loc}} = t_{\mathrm{signal}}^{\eta} + t_{hw}^{\eta}$ (Equations 4-7, main text) the total time needed for a localization with number of cycles η, t_{dwell} the dwell time per localization round as put in the sequence, and N_{PL} the photon limit.

Target Values:

It is useful to have some reference values to compare the results of the MINFLUX experiment. In the case of diffusion measurements, for example, this would be an initial estimate for the diffusion rate $\langle \mathrm{D_{MSD}} \rangle$ taken from previous experiment and/or for simulations. Other kind of dynamics, such as the characteristic motion of motor proteins, are equally helpful.

Tip: It is recommended to examine the EFO histogram during the measurement as follows: The number of Poissonian modes (Optimization Guide Figure 1) provides an approximation of the average number of cycles per localization $\langle \eta \rangle$, which itself provides an estimate of $\langle t_{loc} \rangle$. The variance of the 1-cycle-distribution (See red line in Optimization Guide Figure 1) may be used to gauge photon-use-efficiency $\frac{\langle N \rangle}{\langle \eta \rangle \cdot N_{PL}}$.

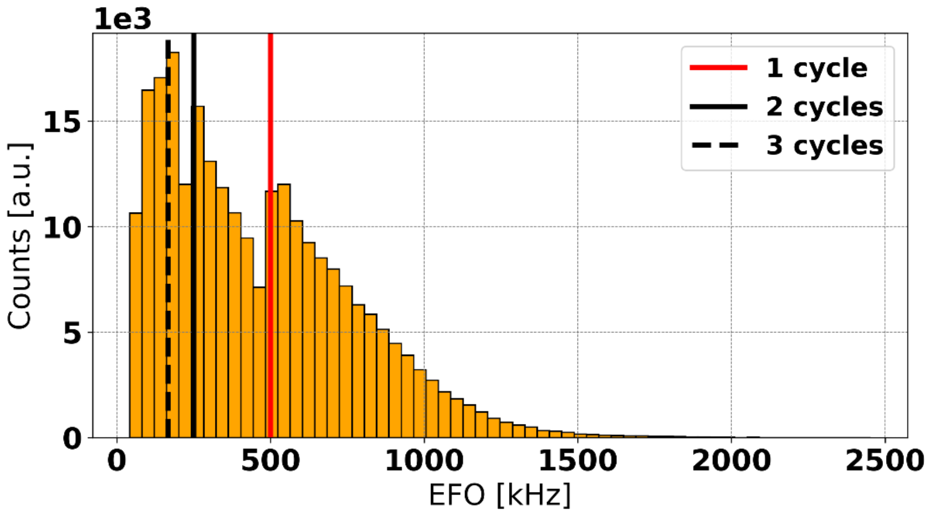

Optimization Guide Figure 1 - Exemplary EFO histogram for the Brownian diffusion of a fluorescent lipid analogue on a GUV-patch SLB (see Materials and Methods) tracked using MINFLUX. Main sequence parameters are $\mathrm{PL} = 50$, $t_{dwell} = 100\mu s$ and $\mathrm{DMP} = 0$ (See Main Text, Table 1). Horizontal lines have been added to visualize the difference between the different number of cycles η. The average number of cycles per localization for this dataset is $\langle \eta \rangle = 2$ (See Main Text, Table 2).

Step 5 Optimization Loop

1. **Perform a MINFLUX experiment and calculate $\langle\boldsymbol{\eta}\rangle$ and $\langle\boldsymbol{t_{loc}}\rangle$.**
2. **If $\langle\boldsymbol{\eta}\rangle = \mathbf{1}$**: *Advance to 3.*
   If $\langle\boldsymbol{\eta}\rangle > \mathbf{1}$: MINFLUX tracking is too slow. Try decreasing the PL or increasing the Power Factor if the sample and background level permit it. *Adjust and start over*.
3. **If $\langle\boldsymbol{t_{loc}}\rangle \leq \boldsymbol{t_{trg}}/\mathbf{2}$** (with t_{trg} the timescale of the effect under study): *Advance to 4*.
   If $\langle\boldsymbol{t_{loc}}\rangle > \boldsymbol{t_{trg}}/\mathbf{2}$: Try lowering the t_{dwell}. Alternatively, although we do not recommend it, consider changing to the triangular TCP pattern which is marginally faster than the hexagonal. *Adjust and start over*.
4. **If $\mathbf{1} < \frac{\langle \boldsymbol{N}\rangle}{\langle\boldsymbol{\eta}\rangle\cdot \boldsymbol{N_{PL}}} < \mathbf{1.5}$**: every photon acquired per cycle is used effectively.
   Advance to 5.
   If $\frac{\langle \boldsymbol{N}\rangle}{\langle\boldsymbol{\eta}\rangle\cdot \boldsymbol{N_{PL}}} > \mathbf{1.5}$: there are photons to spare. Think about increasing the PL or decreasing t_{dwell} to increase the localization precision or tracking speed or consider lowering the Power Factor to decrease photonic stress and potentially increase the track length. *Adjust and start over*.
5. **If reference experiments exist**:
   Calculate and evaluate $\langle D_{MSD}\rangle$:
      If $\langle D_{MSD}\rangle$ matches the expectation, this concludes this optimization scheme.
      If not, it might be necessary to re-evaluate the results of 2. and 3.
   If no reference experiments exist:
      This concludes this optimization scheme.
   Tip: Whether there are reference experiments or not, it is almost always possible to optimize for the localization precision by using the dynamic localization error $\langle\sigma_{\mathrm{MSD}}\rangle$ as target value.

With this optimization loop we have three goals: ensuring fast, homogeneous and reliable tracking by measuring with a single exposure cycle per localization ($\langle\eta\rangle = 1$), and to match the timescale of the experiment as closely as possible while satisfying Nyquist sampling requirements ($\langle t_{loc}\rangle \leq t_{trg}/2$) all while trying to use the photons at our disposal as efficiently as possible increasing the localization precision and reducing photonic stress on the sample $\left(1 < \frac{\langle N\rangle}{\langle\eta\rangle\cdot N_{PL}} < 1.5\right)$.

Tip: If the detected trajectories are extremely short overall, the reason could be that the excitation laser power or Power Factor are too high, bleaching the target very fast, or there could be experiencing focus drift. If the final data set is split between extremely long and extremely short traces, it is more than likely that the MINFLUX sequence is too slow, in which case the results will be heavily biased towards slow and/or immobile particles.

Adjustment Limits

The relevant parameters for optimization of SPT measurements in MINFLUX can be adjusted within the limits below (Optimization Guide Table 1), although it must be kept in mind that any adjustment still needs to be allowed by the set of localization estimation coefficient provided by the manufacturer of the setup.

Optimization Guide Table 1 – Suggestions for the lower and upper limit for the Photon Limit, Dwell Time, and Power Factor *MFX* parameters.

Parameter	Lower Limit	Upper Limit
**Photon Limit**	Equal to the number of vertices of the TCP pattern.	No upper limit.
**Dwell Time**	Equal to the hardware overhead $t_{hw}^{\eta=1}$ ($52\mu s$ for the hexagonal scanning pattern and $35\mu s$ for the triangular pattern).	Half of the relevant timescale of the phenomenon of interest in the experiment, to satisfy Nyquist sampling conditions.
**Power Factor**	1.0, equal to the excitation power input for the experiment.	No upper limit, but photobleaching and other photophysical effects must be kept in mind.

A General Rule of Thumb

The laser power and *PL* should be kept as high as possible and the t_{dwell} as low as possible while maintaining $\langle\eta\rangle = 1$. That way the time-to-localization is automatically minimized, while maximizing lateral localization precision and tracking rate.

A Note on Optimizing Non-Final Pattern Iterations within the *MFX*-Sequence

The commercial implementation of MINFLUX microscopy (*Abberior Instruments* GmbH.) is shipped with a variety of sequences corresponding to different use cases (imaging and tracking sequences), which include varying numbers of *pattern iterations* with diminishing values of L. In our experience, iterations before the last rarely require optimization from the default, since the only purpose they serve is to identify candidate emission events to localize a single fluorophore as quickly as possible. More weight is usually put on the final iteration as it produces the final localization estimates and, consequently, single molecule trajectories. Nevertheless, it is important to be aware of these *pattern iterations*, and of the possibility of optimizing them in the same way as the final one, using the protocol exposed above.

One instance in which optimization of previous *pattern iterations* may be considered is in case no localizations are detected despite the sample having noticeable fluorescence. This is often caused by other factors, such as insufficient fluorescence excitation, too high fluorescence excitation (resulting in premature bleaching of the detected fluorophore), or overestimation of the background threshold (which is a parameter of the sequence). If these have been optimized, but no improvement is observed, other issues may be at play, such as exceedingly high density of fluorophores, resulting in *MFX* being unable to single out and localize particles, or diffusing targets which are too fast for the device to track in spite of optimization efforts.
