# Supplementary material for: Parameter optimization for MINFLUX microscopy enabled single particle tracking
Source: Commun Biol. 2025 Nov 14;8:1573. doi: 10.1038/s42003-025-09060-1 (PMC12618476; doi:10.1038/s42003-025-09060-1)
Supplement: Supplementary file 2 — Reporting Summary [file 42003_2025_9060_MOESM2_ESM.pdf]

nature portfolio

Corresponding author(s): Prof. Dr. Christian Eggeling

Last updated by author(s): Sep 18, 2025

Reporting Summary

Nature Portfolio wishes to improve the reproducibility of the work that we publish. This form provides structure for consistency and transparency in reporting. For further information on Nature Portfolio policies, see our Editorial Policies and the Editorial Policy Checklist.

Statistics

For all statistical analyses, confirm that the following items are present in the figure legend, table legend, main text, or Methods section.

n/a	Confirmed	
☐	☒	The exact sample size (*n*) for each experimental group/condition, given as a discrete number and unit of measurement
☐	☒	A statement on whether measurements were taken from distinct samples or whether the same sample was measured repeatedly
☒	☐	The statistical test(s) used AND whether they are one- or two-sided<br>*Only common tests should be described solely by name; describe more complex techniques in the Methods section.*
☒	☐	A description of all covariates tested
☒	☐	A description of any assumptions or corrections, such as tests of normality and adjustment for multiple comparisons
☐	☒	A full description of the statistical parameters including central tendency (e.g. means) or other basic estimates (e.g. regression coefficient) AND variation (e.g. standard deviation) or associated estimates of uncertainty (e.g. confidence intervals)
☒	☐	For null hypothesis testing, the test statistic (e.g. *F*, *t*, *r*) with confidence intervals, effect sizes, degrees of freedom and *P* value noted<br>*Give P values as exact values whenever suitable.*
☒	☐	For Bayesian analysis, information on the choice of priors and Markov chain Monte Carlo settings
☒	☐	For hierarchical and complex designs, identification of the appropriate level for tests and full reporting of outcomes
☒	☐	Estimates of effect sizes (e.g. Cohen's *d*, Pearson's *r*), indicating how they were calculated

Our web collection on statistics for biologists contains articles on many of the points above.

Software and code

Policy information about availability of computer code

Data collection	MINFLUX IMSPECTOR (commercial version - 16.3.15645-m2205), TIRF Microscope (custom) & Control Software (custom)
Data analysis	Custom Code to:<br>- read, filter, display data<br>- extract the MINFLUX time signal 2D<br>- homogenize the MINFLUX time signal (2D)<br>- calculate Mean Squared Displacement for inhomogeneous time signal<br>- extract diffusion rates and anomalous diffusion coefficients<br>- calculate and plot (Cramer-Rao Lower Bound, TCP area coverage)

For manuscripts utilizing custom algorithms or software that are central to the research but not yet described in published literature, software must be made available to editors and reviewers. We strongly encourage code deposition in a community repository (e.g. GitHub). See the Nature Portfolio guidelines for submitting code & software for further information.

Data

Policy information about availability of data

All manuscripts must include a data availability statement. This statement should provide the following information, where applicable:

- Accession codes, unique identifiers, or web links for publicly available datasets
- A description of any restrictions on data availability
- For clinical datasets or third party data, please ensure that the statement adheres to our policy

All data and sequences used in the manuscript are made available withing the Parameter-Optimization-for-MINFLUX-Microscopy repository on GitHub (https://github.com/Zatyrus/Parameter-Optimization-for-MINFLUX-Microscopy). The provided data includes the raw information extracted from the proprietary file structure (Abberior Instruments GmbH) returned by the MINFLUX in a streamlined format, the raw TIRF SPT trajectories, the processed MINFLUX and TIRF SPT trajectories including MSD and metadata, and the numerical source data for all graphs and charts found in this work. Files are named based on their respective experimental parameters. We include a toolbox to conveniently access and format the data.
The raw MINFLUX files as exported from MINFLUX-iMSCPECTOR (commercial version - 16.3.15645-m2205) as well as the raw TIRF image stacks are made available within a Zenodo repository (Data Deposition - Parameter Optimization for MINFLUX Microscopy enabled Single Particle Tracking; doi: 10.5281/zenodo.17153525).

Research involving human participants, their data, or biological material

Policy information about studies with human participants or human data. See also policy information about sex, gender (identity/presentation), and sexual orientation and race, ethnicity and racism.

Reporting on sex and gender	n.a.
Reporting on race, ethnicity, or other socially relevant groupings	n.a.
Population characteristics	n.a.
Recruitment	n.a.
Ethics oversight	n.a.

Note that full information on the approval of the study protocol must also be provided in the manuscript.

Field-specific reporting

Please select the one below that is the best fit for your research. If you are not sure, read the appropriate sections before making your selection.

☒ Life sciences ☐ Behavioural & social sciences ☐ Ecological, evolutionary & environmental sciences

For a reference copy of the document with all sections, see nature.com/documents/nr-reporting-summary-flat.pdf

Life sciences study design

All studies must disclose on these points even when the disclosure is negative.

Sample size	The relevant definition of sample size for this work is determined by the number of trajectories present within a single dataset.
Data exclusions	We did not exclude any data preemptively. However, we used several routines for artifact detection and removal as well as time signal homogenization. All of which we briefly describe or cite in the manuscript.
Replication	We have replicated data by recording several datasets on the same and different samples.
Randomization	n.a.
Blinding	n.a.

Reporting for specific materials, systems and methods

We require information from authors about some types of materials, experimental systems and methods used in many studies. Here, indicate whether each material, system or method listed is relevant to your study. If you are not sure if a list item applies to your research, read the appropriate section before selecting a response.

Materials & experimental systems

n/a	Involved in the study
☒	☐ Antibodies
☒	☐ Eukaryotic cell lines
☒	☐ Palaeontology and archaeology
☒	☐ Animals and other organisms
☒	☐ Clinical data
☒	☐ Dual use research of concern
☒	☐ Plants

Methods

n/a	Involved in the study
☒	☐ ChIP-seq
☒	☐ Flow cytometry
☒	☐ MRI-based neuroimaging

Plants

Seed stocks	n.a.
Novel plant genotypes	n.a.
Authentication	n.a.
